# Epigenetics Control Microglia Plasticity

**DOI:** 10.3389/fncel.2018.00243

**Published:** 2018-08-03

**Authors:** Mathilde Cheray, Bertrand Joseph

**Affiliations:** Toxicology Unit, Institute of Environmental Medicine, Karolinska Institutet, Solna, Sweden

**Keywords:** microglia, epigenetics, histone post-translational modification, DNA methylation, non-coding RNAs

## Abstract

Microglia, resident immune cells of the central nervous system, fulfill multiple functions in the brain throughout life. These microglial functions range from participation in innate and adaptive immune responses, involvement in the development of the brain and its homeostasis maintenance, to contribution to degenerative, traumatic, and proliferative diseases; and take place in the developing, the aging, the healthy, or the diseased brain. Thus, an impressive level of cellular plasticity, appears as a requirement for the pleiotropic biological functions of microglia. Epigenetic changes, including histone modifications or DNA methylation as well as microRNA expression, are important modifiers of gene expression, and have been involved in cell phenotype regulation and reprogramming and are therefore part of the mechanisms regulating cellular plasticity. Here, we review and discuss the epigenetic mechanisms, which are emerging as contributors to this microglial cellular plasticity and thereby can constitute interesting targets to modulate microglia associated brain diseases, including developmental diseases, neurodegenerative diseases as well as cancer.

## Introduction

Microglia are the immune cells of the brain, they derived from myeloid precursors which migrate into the brain during early embryonic development and play a major role in maintaining a healthy environment in the brain (Ginhoux et al., 2010; Prinz and Mildner, 2011). Microglia are constantly screening the brain environment by using their surface receptors to detect damaged neurons, plaques, and infectious agents. In this steady or surveying state, microglia presents a high amount of ramified processes that perform constant screening of the central nervous system (CNS) environment. When activated by a stimulus, microglia can act as potent immune cells able to mediate innate and adaptive responses and to perform different function during CNS disease or injury. Microglia are of main importance for the brain homeostasis but uncontrolled or over-activated microglia can also contribute to brain diseases. Over-activated microglia can promote neuronal cell death in the course of neurodegenerative pathologies like Alzheimer’s disease (AD), Parkinson’s disease (PD), Huntington’s disease (HD) as well as amyotrophic lateral sclerosis (ALS) (Sarlus and Heneka, 2017). Uncontrolled microglia will also lead to increase tumor cell migration and invasion in the case of brain tumor (Saijo and Glass, 2011).

Some of the main characteristics of microglia is their ability to adapt to their microenvironment and to acquire a specific phenotype based on stimuli they perceive. As innate immune cells they will become activated in response to injury, infection or death of cell in their vicinity (Eggen et al., 2013). Microglia can harbor different phenotypes depending of the signal they receive/detect from the microenvironment. Since microglia are frequently define as the macrophages of the brain, their classification and phenotypes have been established and named based on macrophages characteristics. Microglia will be activated in a pro-inflammatory phenotype or so called classical activation (M1 phenotype) in response to viral or bacterial infection, in the opposite, microglia will harbor an anti-inflammatory phenotype or alternative activation (M2 phenotype) in case of neurodegenerative diseases when detecting neuronal cell death for example. Worth a notice, the concept of the microglial M1/M2 polarization has been heavily questioned. In fact, it is now recognized that microglia display a continuum of activation states, which are largely oversimplified in the M1/M2 classification. However, since the M1/M2 paradigm has been heavily used in the literature, and for simplicity, references are made in this review to microglial M1 and M2 phenotypes. Recently, it has also been shown that microglia can acquire an alternative phenotype, or pro-tumoral phenotype, where the microglia cells are involved in increasing tumor invasiveness (Hambardzumyan et al., 2016). With the discovery of the ability of microglia to support tumor growth, it was shown that microglia is able to adapt to the microenvironment and its activation process and phenotype acquisition is more complex than thought. Indeed, microglia will harbor different transcriptional factor activation signatures depending on the phenotype they will acquire (e.g., neuroprotective, lipid response, neuropathic pain) which confirm the extreme plasticity of these cells and the complexity of the microglia phenotypical changes (Holtman et al., 2017). Thus, microglia fulfill multiple and contrasting functions across development and adulthood, in steady-state conditions, but also in the context of diseases. To perform their multiple functions, microglia appear to exhibit various phenotypes. Microglia thus appear to display extreme plasticity, and are able to modify their structure and functions based on their role and location. But, how can microglia obtain this level of cell plasticity?

In response to different stimuli, cells can change their state of activation and adapt their identity. These modifications are regulated by finely tuned modulation of gene expression. This coordinated regulation is mainly a result of changes in the composition and the structure of the chromatin, driven by epigenetic modulators. Recently, it has been shown that cellular and genomic reprogramming is highly dependent of chromatin modifications in order to lead and maintain the fidelity of target cell states. Epigenetic modifications such as histone modifications (e.g., methylation, acetylation, phosphorylation), DNA methylation or gene expression regulation by non-coding RNAs are crucial for normal development but can also be involved in diseases (Esteller, 2008). Epigenetic modifications do not modify the genome *per se* of the cell but they do regulate gene expression and thereby contribute to the definition of cell phenotype. For example, DNA methylation patterns, which are reversible heritable marks conserved during cell division, are involved in cell reprogramming processes (De Carvalho et al., 2010). Epigenetic modifications have been involved in cell phenotype regulation like the reprogramming of stem cells. Indeed, stem cells have the ability to self-renew or differentiate and these changes in cell phenotype involve a fine-tuned regulation by epigenetic mechanisms (Chung and Sidhu, 2008; Zhou et al., 2011; Katsushima and Kondo, 2014). Microglia can be compared to stem cells in their ability to adapt to the microenvironment and to differentiate into a specific cell phenotype in response to the signal activation. Thus, these cells are very plastic, however, until now, even if microglia have been known for almost a century, the mechanisms leading to their activation toward a specific phenotype are not yet fully established, but it seems obvious that epigenetic changes should contribute to the microglia plasticity.

Here, we review the roles of epigenetic alterations, including histone modifications, DNA methylation or microRNA expression, as well as the enzymatic systems regulating those modifications, may have in regulating microglia plasticity and polarization toward unique phenotypes. We also consider the contribution of the epigenetic control of microglia to their activation states in the context of health and disease, and possible long-term and lasting microglial effects such as the one observed upon microglial priming/memory or even transgenerational microglial effects.

## Histone Modifications in Microglia

The structure of the chromatin is regulated by its compaction depending on the organization of the histone proteins. The chromatin is composed of the DNA, tightly packed around histone proteins organized in units called nucleosomes. A nucleosome is composed by two H3-H4 dimers surrounded by two H2A-H2B dimers corresponding to an octomeric core of histone proteins. Histone tails amino-terminal parts are protruding from the nucleosome, which make them accessible for possible post-translational modifications. Depending on the nucleosome spacing, the chromatin structure will be defined as heterochromatin and euchromatin. Heterochromatin corresponds to a condensed state of the chromatin while euchromatin is non-condensed or open chromatin. This open state of the chromatin allows the nuclear factors to access the chromatin. Modifications occurring on the histone tails and on the DNA are involved in the regulation of the chromatin structure and of the gene accessibility by the transcriptional machinery. Histones can be methylated, acetylated or phosphorylated on specific amino acids residues located on the histone tails. The chromatin accessibility is altered by histone acetylation which allow the interaction of DNA binding protein to accessible sites in order to activate gene transcription. Histone acetylation is carried out by histone acetyl transferases (HATs), which acetylate the lysine residues on histones tails or core, on the converse, the role of histone deacetylases (HDACs) is to remove the acetyl groups from those lysine residues. Histone methylation is either associated to transcription activation or repression depending on which amino acid the modification occurs. Histone methyltransferases (HMTs), promote the mono- di- or tri-methylation on target histone residues, whereas histone demethylases (HDMs) counteract the effects of the HMTs.

The potential use of HDAC inhibitors in inflammatory/neurodegenerative diseases has been extensively investigated, since histone acetylation was shown to regulate the extent of inflammatory response (Blanchard et al., 2002; Ito et al., 2002). In the recent years, HDAC inhibitors have been widely used to target microglia with the aim of reducing inflammation. Valproic acid (VPA), defined as a non-selective HDAC inhibitor, is a FDA approved drug used to treat epilepsy and bipolar disorders. In the context of spinal cord injury, the ability of this drug to reduce the inflammatory response after injury and to avoid the appearance of exacerbating pathogenic events was assessed. In their study, Abdanipour et al. (2012) observed a reduction of the local inflammation and a reduction of microglia activation (as illustrated by reduction of the ED1 lysosomal marker), which was associated with an improvement in the animal, behavior, in rats treated with VPA after the injury. The use of VPA to target microglia has also been studied in the context of inflammation and innate antiviral gene expression (HIV for example) using a model of primary human microglia and astrocytes treated with TLR3 or TLR4 ligand. VPA has a direct effect on microglia since it suppresses the expression of chemokine and cytokine gene expression, of innate antiviral molecules (like IFNβ) and of protein related to the activation of the TLR3-TLR4 signaling pathway (Suh et al., 2010).

The potential use of TSA to target microglia has also been widely explored. Its effect was tested on lipopolysaccharide (LPS)-induced inflammatory response in microglia. TSA treatment has been described first as strongly potentiating the inflammatory response induced by LPS treatment in different models: from the N9 cell line and primary microglia cells to hippocampal slices culture as well as in neural co-cultures (Suuronen et al., 2003). More recently, TSA has been defined as a suppressor of the inflammatory phenotype of microglia after LPS treatment. Indeed, HDAC inhibition by TSA or SAHA (also known as Vorinostat) leads to a suppression of cytokine expression and release after LPS induction in primary microglia cultures (Kannan et al., 2013), but also in the mouse brain where cognitive dysfunction induced by LPS (weight loss, anorexia, and social withdrawal) was also attenuated (Hsing et al., 2015). Moreover, hyperacetylation induced by TSA treatment has a neuroprotective effect in the female neonatal mouse after LPS and hypoxia-ischemia and correlates with an improvement of long-term learning (Fleiss et al., 2012).

As VPA and TSA, sodium butyrate (SB) has also been studied for its potential role in inflammation regulation through microglia targeting. In this matter, it has been shown that SB induces changes in microglia shape, with apparition of microglial processes elongation in inflammatory and normal conditions associated with changes in pro-inflammatory and anti-inflammatory microglia markers (Wang et al., 2018). Patnala et al. (2017) also observed an alteration of the H3K9ac enrichment and transcription at the promoters of genes related to microglia activation (*Tnf-α*, *Nos2*, *Stat1*, *IL6*, and *IL10*) after SB treatment. In a microglia model of middle cerebral artery occlusion (MCAO), an upregulation of H3K9ac levels was observed, which links H3K9ac upregulation to microglial activation *in vivo*. The same observation was made in post-seizure microglia cells where SAHA pre-treatment reduces the levels of H3 and H3K9 acetylation to baseline levels (Hu and Mao, 2016).

Inhibitors of HDACs decrease the inflammatory response of isolated microglia exposed to inflammogens such as LPS. HDAC inhibitors treatment also produces a rapid and sustained increase in the level of histone H4 acetylation in microglia (Durham et al., 2017). Likewise, specific knockdown of *Hdac1* and *Hdac2* using siRNA was found to reduce LPS-induced microglia activation *in vitro* (Durham et al., 2017). In the context of regulating the inflammatory response of microglia, these two HDACs show functional redundancy, as an upregulation of HDAC2 expression appears to be able to compensate for HDAC1 deficiency. Of note, these two HDACs are reported to exert both distinct and redundant functions. In fact, with few exceptions, targeted deletion of either *Hdac1* or *Hdac2* does not cause obvious phenotype in most tissues or cell types (Kelly and Cowley, 2013). Recently, Datta et al. (2018), taking advantage of *Cx3cr1Cre Hdac1^fl/fl^Hdac2^fl/fl^* mouse model, demonstrated that the combined *Hdac1* and *Hdac2* gene depletion *in vivo* differentially affected microglia during development, homeostasis and during neurodegeneration. Indeed, whereas *Hdac1-2* deletion in adult microglia has no effect on cell number or morphology during homeostasis, in the course of neurodegeneration such as in an AD mice model, *Hdac1-2* deletion positively affected microglial phagocytosis of amyloid plaques. Identical genetic intervention in microglia prior to birth also strongly impaired on their development, and resulted in reduced cell number and altered morphology (Datta et al., 2018). Transcriptome analysis revealed that at birth 4,338 genes were differentially expressed in *Hdac1-2* null mice as compared to wild-type mice, the number of differentially regulated genes appeared to decrease overtime, suggesting that HDAC1 and HDAC2 are key players for microglia development.

Genome-wide profiling for histone H3 lysine K9 and K27 acetylation, revealed that the global levels of H3K9ac and H3K27ac are not significantly affected in microglia lacking Hdac1 and Hdac2. Only a deeper inspection revealed that the abundance of H3K9ac and H3K27ac are increased at the proximal promoters of genes regulating cell cycle and cell activation, e.g., *Sema6d*, *Cdkn2c*, *Ifnar2*, and *Kcna3* (Datta et al., 2018). Thus, which histone post-translational modification(s) may account for the significant transcriptional effects of *Hdac1-2* deletion in microglia remain to be identified. This identification is a challenge as there is no direct binding of HDAC1/2 to the DNA and also because those proteins are part of several multiprotein complexes in the nucleus i.e., the NuRD (nucleosome remodeling and deacetylation), the Sin3 and the CoREST (co-repressor for element-1-silencing transcription factor) complexes (Kelly and Cowley, 2013).

**FIGURE 1:**
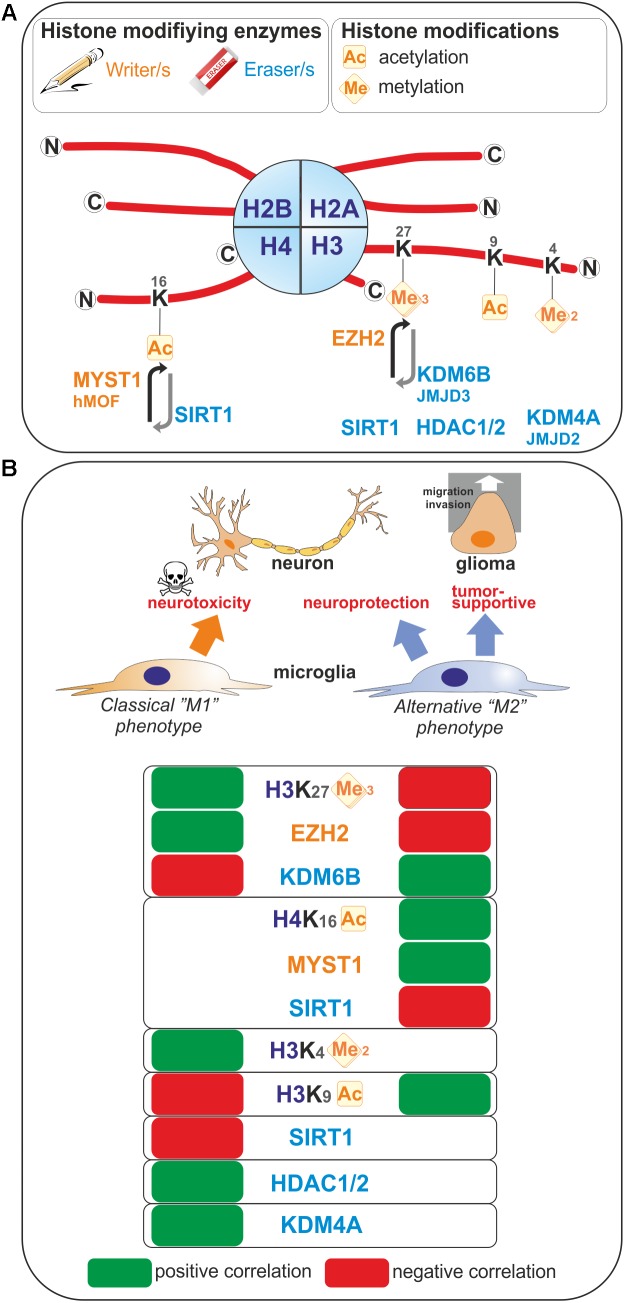
Histone modifications occurring upon microglia polarization. **(A)** Post-translational modifications on histone tails that occur in microglia are represented. These covalent modifications are added or removed by histone modifying enzymes often referred as writers and erasers. **(B)** Positive or negative correlation for the expression levels of these histone modifications or the enzymes regulating those histone marks with the polarization toward so-called “M1” or “M2” microglia phenotypes are reported. Ac, Acetylated; me, methylated.

Enhancer of zeste homolog 2 (EZH2) histone methyltransferase and the catalytic subunit of the Polycomb repressive complex 2 mediate transcriptional silencing through the tri-methylation of the lysine 27 in histone H3 (H3K27me3). The histone H3K27me3 demethylase Jumonji domain containing 3 (JMJD3, also known as KDM6B) counteracts the effect of EZH2, and therefore the H3K27me3 levels are regulated by the balance between activities of EZH2 and JMJD3. In microglia, *EZH2* gene expression is found to be significantly and rapidly increased upon a pro-inflammatory stimulus such as LPS-mediated TLR4 stimulation (Arifuzzaman et al., 2017; Zhang et al., 2018). Remarkably, JMJD3 expression is found to be up-regulated upon IL-4 treatment which promotes alternative microglial activation (Tang et al., 2014), but as well LPS-treatment (Lee et al., 2014; Das et al., 2017). These observations suggest that M1- and M2-stimuli differentially regulate microglial H3K27me3 expression level, and thereby these microglia activation states (**Figure 1**). In fact, inhibition of EZH2 with the selective small molecule inhibitors EPZ-6438, GSK343, or GSK126 suppressed the expression of genes mediating the pro-inflammatory response (Koellhoffer et al., 2014, 2015; Arifuzzaman et al., 2017; Zhang et al., 2018). Moreover, inhibition of EZH2 with GSK343 in the presence of an IL-4 stimulus results in a significant upregulation of M2-related genes (Koellhoffer et al., 2014, 2015). Furthermore, *Ezh2* deficiency in microglia *in vivo*, achieved by crossing *Ezh2* floxed mice with Cx3cr1-CreER mice, confirmed in an animal model of experimental autoimmune encephalomyelitis, that EZH2 facilitates activation of microglia toward a pro-inflammatory M1 phenotype (Zhang et al., 2018). Mechanistically, EZH2 has been proposed to directly target and repress the expression of gene encoding for suppressor of cytokine signaling 3 (SOCS3), known to promote the proteosomal degradation of tumor necrosis factor receptor-associated factor 6 (TRAF6), key component of the TLR-induced MyD88-dependent NF-kB activation contributing to acquisition of the microglial pro-inflammatory phenotype (Zhang et al., 2018). In addition, EZH2 also appears to regulate positively the expression of the transcription factors interferon regulatory factor (IRF) 1, IRF8, and signal transducer and activator of transcription 1 (STAT1), which have known roles in regulating inflammation (Arifuzzaman et al., 2017). In summary, the H3K27 histone tri-methyltransferase activity of EZH2 promotes M1 microglia polarization but represses M2 microglia polarization.

In contrast, the histone H3K27me3 demethylase activity of JMJD3 appears to promote M2 microglia polarization but represses M1 microglia polarization. At first, the observation that JMJD3 expression is found to be increased in microglia upon stimulation with an inflammogen may sound contradictory to a role in repressing a pro-inflammatory activation of these cells. However, if one considers a negative-regulatory loop induced upon M1 stimulation, the effects of JMJD3 can be explained and supported by the existing literature. Indeed, *JMJD3* depletion or inhibition in microglia leads to an inhibition of the polarization of microglia into a M2 phenotype and increases the M1 inflammatory response of microglia *in vitro* (Tang et al., 2014; Das et al., 2017). In addition, dehydroepiandrosterone, one the most abundant circulating steroid hormone in humans, is reported to inhibit LPS-induced microglia-mediated inflammation by further increasing JMJD3 expression level above the one induced by LPS treatment alone (Alexaki et al., 2017), suggesting that JMJD3 expression levels are of importance in regulating microglia polarization. Interestingly, knockdown of *Jmjd3* gene *per se* is sufficient to compromise the expression of various M2 genes, including *Arg1*, *CD206*, and *Igf1* genes, suggesting that the regulation of microglia M2 polarization by JMJD3 is an intrinsic and cell autonomous mechanism. Suppression of JMJD3 expression *per se* also induces an exaggerated polarization of microglia into M1 phenotype as illustrated by the expression of genes of pro-inflammatory factors including *iNOS*, *IL1β*, and *IL6*. Finally, *in vivo*, in an animal model of PD, the suppression of *Jmjd3* in the substantia nigra was shown to promote microglial over-activation and thereby exacerbate dopamine neuron loss (Tang et al., 2014). Of note, controversial observation of JMJD3 role has been observed in microglia. Indeed, by transcriptional sequencing analysis of the effect of the JMJD3 inhibitor GSK-J4 on primary microglia cells and BV2 microglia cells, Das et al. (2017) showed that this inhibitor have a selective effect on the expression of genes induced by LPS since it suppresses the induction of chemokines and cytokines, of transcription factors and interferon-stimulated genes. Moreover, it is shown that STAT1 and STAT3 are regulating JMJD3 transcription and cooperates with this transcription factor to induce pro-inflammatory genes expression (Przanowski et al., 2014).

The H3K9ac histone mark which, when located near the transcription start site is essentially related to transcription activation, has been linked to microglia activation (**Figure 1**). Indeed, in primary and BV2 microglia cells, acetate treatment induces H3K9 hyperacetylation, reverses LPS-induced H3K9 hypoacetylation (Soliman et al., 2012) and increases the amount of acetylated H3K9 bound to the promoter regions of *Cox1*, *Cox2*, *IL1β*, and *NFκB p65* genes (Soliman et al., 2013). An increase of H3K9ac has also been found in microglia in a model of neuropathic pain. In this model, the authors study the benefit of exercise on neuropathic pain. By investigating the nuclear expression of H3K9ac in microglia, it has been shown that, in a mice model of partial sciatic nerve ligation (PSL), running exercise increased significantly the number of microglia expressing acetylated H3K9 compared to sedentary mice suggesting a role of H3K9 acetylation in producing exercise-induced hypoalgesia (Kami et al., 2016).

Methylation of histone 3 on lysine 4 residue is linked to active transcription. H3K4me2 is defined as a specific mark for promoters and enhancers (He et al., 2010; Kaikkonen et al., 2013), while acetylation of H3K27 corresponds to their transcriptional activity (Creyghton et al., 2010). By analysis of the transcriptomes and enhancer landscapes of resident macrophages and microglia using ATAC-seq and ChIP-seq approaches, Gosselin et al. (2014) revealed the existence of specific pattern for H3K4me2 deposition in microglia, which substantially differs from that of large peritoneal macrophages. Moreover, by identifying a new type of microglia, Disease associated microglia or DAM, Keren-Shaul et al. (2017) suggested a role of H3K4me2 in the priming of microglia. Indeed, by using a specific method of ChIP-sequencing with high sensitivity and comparing enhancers from DAM and microglia in wild-type or Alzheimer disease mouse model, they observed a highly similar global pattern of H3K4me2, which is present at promoters and enhancers regions. For DAM-specific genes, the authors also observed that active H3K4me2 regions are present in DAM but also in microglia. This suggests that homeostatic microglia already contains the DAM program (Keren-Shaul et al., 2017).

In opposition to the JMJD family, the histone lysine demethylases enzymes (KDM) catalyze the removal of methyl marks from histone lysine residues which leads to the regulation of the structure of the chromatin and the gene expression by epigenetic changes. By LPS treatment activation, BV2 cells and primary microglia cells showed an upregulation of the JMJD2 (also known as KDM4A) and KDM1A enzymes, respectively (Das et al., 2015b, 2016). Moreover, stimulation of microglia cells by TLR3- and TLR4 ligands results in an upregulation of the JMJD2 enzyme in the BV2 microglia cell line (Das et al., 2015a).

Little is known concerning histone phosphorylation in microglia. One study focused on Endocannabinoids which are supposed to reduce neuronal damage after their release in the case of brain injury. In this context, Eljaschewitsch et al. (2006) showed that in microglia, the cannabinoid receptor pathway (CB1/2) and the production of MKP-1 (via the phosphorylation of Histone H3) lead to the suppression of *iNOS* expression and NO production. The authors conclude that the AEA endocannabinoid activates the phosphorylation of the histone H3 and subsequently the expression of MKP-1 which will lead to block the release of NO only in microglia treated with LPS, leading to neuroprotection (Eljaschewitsch et al., 2006).

Sirtuin 1 (SIRT1) is involved in different cellular processes like inflammation or aging/senescence (Gan and Mucke, 2008; Libert and Guarente, 2013). SIRT1 acts as a deacetylase enzyme with different intracellular targets like histones among others (Michan and Sinclair, 2007; Zhang et al., 2011). Cho S.H. et al., 2015 reported a reduction of SIRT1 with the aging of microglia. This SIRT1 reduction can be associated with aging but also with memory deficits mediated by Tau via the upregulation of IL-1β in mice (Cho S.H. et al., 2015). Moreover, we recently studied the epigenetic changes occurring in microglia under the influence of brain tumor cells (glioma cells). By using a coculture system between microglia and glioma cells we observed that the activation of microglia by glioma cells induces an increase of H4K16ac in microglia. This is due to the increase of SIRT1 into the nucleus of microglia cells which will deacetylates hMOF (a H4K16 acetyltransferase) leading to its chromatin recruitment at the promoter of specific microglia target genes (Saidi et al., 2018; **Figure 1**).

## Microrna and Long Non-Coding Rna

Non-coding RNAs (ncRNAs), including long non-coding RNAs (lncRNAs) and microRNAs (miRNAs/miRs) serve important roles in regulating the expression of certain genes (Patil et al., 2014) and have emerged as epigenetic regulators of biological processes in microglia.

MicroRNAs are small (20–30 nucleotides in length), highly conserved ncRNAs that regulate gene expression post-transcriptionally. They bind to the 3′-UTR (untranslated region) of their mRNAs target(s) and thereby downregulate gene expression through the RNA interference pathway. Worth a notice, miRNAs are involved in the fine-tuning regulation of the expression of around 30% of all mammalian protein-encoding genes.

In fact, microglial steady-state as well as the different microglia activation states can be characterized by distinct miRNA signatures. Indeed, Butovsky et al. (2014) uncovered, using a Nanostring based miRNA chip that contains 600 microRNAs, that eight of these microRNAs, i.e., miR-29a, mIR-29b, miR-30a, miR-99a, miR-103, miR-125b, miR-322, and miR-342 are highly expressed in unchallenged mouse microglia. Furthermore, three of those (miR-99a, miR-125b, and miR-342) were found to have a microglial specific expression compared to other immune cells (Butovsky et al., 2014).

The role of miRNAs in regulating the microglial activation has been investigated in the context of multiple stimuli, brain challenges and diseases. For simplicity, the M1/M2 paradigm is used in this review when reporting the effect of various miRNAs on microglial activation states. The current literature does not allow for most of the reports a more detailed definition of the microglia phenotypes. In order to elucidate the role miRNAs may exert on the acquisition of the M1 versus M2 microglial phenotypes, Freilich et al. (2013) performed miRNAs expression profiling on primary murine microglia exposed to lipopolysaccharide (M1-like condition) or interleukin-4 (M2-like condition). Using a mouse miRNA Array that interrogates 690 pre-miRNAs and 722 mature miRNAs, they revealed that: (1) upon lipopolysaccharide stimulation of microglia, 12 miRNAs were increased and 35 were reduced; (2) upon interleukin-4 stimulation of microglia, 16 miRNAs were increased and 28 were decreased. In this study, miR-155 and miR-145 were identified as the most significantly up-regulated miRNAs in the context of lipopolysaccharide-stimulation of microglia and interleukin-4 -stimulation of microglia, respectively. The differentiation regulation of miR-155 and miR-145 expression in microglial M1 versus M2 phenotypes, has been confirmed in the context of other stimuli promoting those phenotypes (Cardoso et al., 2012; Woodbury et al., 2015; Qi et al., 2017; Xie et al., 2017; Yin H. et al., 2017). The microRNA miR-155 has gained particular attention in the context of ALS. Indeed, in the *SOD1^G93A^* mouse model of ALS, overexpressing the human *SOD1* gene carrying a glycine to alanine point mutation at residue 93 (G93A), increased microglial miR-155 expression in the pre-symptomatic mice suggests that this miR could be used as a marker to track ALS at an early stage. In contrast, other microRNAs, such as miR-125b, miR-146a, and miR-124, were only found to be upregulated at the symptomatic stage (Cunha et al., 2018). Furthermore, targeting miR-155 appears to restore microglia function and prolongs survival of the *SOD1^G93A^* mice (Koval et al., 2013; Butovsky et al., 2015). Further, significant down-regulation of miR-689 was found to associate with the M1-activation phenotype, whereas down-regulation of miR-711 associated with the M2-activation phenotype. Remarkably, reduced miR-124 expression was observed upon activation toward both phenotypes suggesting that miR-124 expression may be associated to the microglia steady-state and thus to be added to the miRNAs microglial signature described by Butovsky and coworkers (Butovsky et al., 2014).

Of the potential epigenetic regulators that can control microglia biology, miRNAs are probably the most investigated one. Illustration of these considerable efforts are depicted in **Table 1** that report the different miRNAs, as well as their target genes, which have been reported to affect microglia activation.

**Table 1:** MicroRNAs regulating microglia activation and their gene targets.

Function	miRNA	Gene target	Reference
Negative regulator of microglial pro-inflammatory activation	miR-7	*NLRP3*	Zhou Y. et al., 2016
	miR-26a	*ATF2*	Kumar et al., 2015
	miR-27a	*TLR4, IRAK4*	Lv et al., 2017
	miR-30a	*NEUROD1*	Fu et al., 2018
	miR-93		Tian et al., 2017
	miR-124	*RFX1*	Dong et al., 2016; Guo et al., 2016; Hamzei Taj et al., 2016a; Feng et al., 2017; Cunha et al., 2018
	miR-125b	*STAT3*	Parisi et al., 2013; Cunha et al., 2018
	miR-128		Yang et al., 2017
	miR-145	*PLA2G4A*	Qi et al., 2017
	miR-146a		Sharma et al., 2015; Zhou et al., 2015; Zhao et al., 2016; Deng et al., 2017; Cunha et al., 2018
	miR-181c	*MLL1*	Ma et al., 2016; Yin M. et al., 2017
	miR-199b	*IKKβ*	Zhou et al., 2016
	miR-200b	*JUN*	Jadhav et al., 2014
	miR-203	*MYD88*	Yang et al., 2015
	miR-339	*IKKβ, IKKe*	Zhang et al., 2014
	miR-365	*IL6*	Parisi et al., 2013
	miR-367	*IRAK4*	Yuan et al., 2015
	miR-424	*CDC25A, CCND1, CDK6*	Zhao et al., 2013; Liu et al., 2015
	miR-Let-7a		Cho K.J. et al., 2015
	miR-Let-7c	*CASP3*	Lv et al., 2018
Positive regulator of microglial pro-inflammatory activation	miR-9	*MCPIP1*	Yao et al., 2014
	miR-29b	*TNFAIP3*	Thounaojam et al., 2014
	miR-145	*NR4A2*	Xie et al., 2017
	miR-155	*RACK1, SOCS-1*	Cardoso et al., 2012; Butovsky et al., 2015; Woodbury et al., 2015; Yin H. et al., 2017
	miR-195	*ATG14*	Shi et al., 2013
	miR-204	*SIRT1*	Li et al., 2015
	miR-206	*IGF1*	Xing et al., 2016
	miR-221	*SOCS1*	Xia et al., 2016
	miR-7116	*TNFα*	He et al., 2017
Positive regulator of microglial anti-inflammatory activation	miR-124	*RAC1*	Brennan et al., 2016; Hamzei Taj et al., 2016b; Louw et al., 2016; Svahn et al., 2016
	miR-Let-7a		Song et al., 2015
	miR-Let-7c	*CASP3*	Ni et al., 2015

In addition to miRNAs, lncRNAs, which are a type of ncRNAs that exceed 200 nucleotides in length, have also emerged as potential factors contributing to the regulation of microglia activation states. lncRNAs H19, MALAT1, Gm4419, and SNHG14 have been reported to stimulate the activation of microglia toward the M1 phenotype and thereby promote neuroinflammation (Qi et al., 2017; Wang et al., 2017; Wen et al., 2017; Han et al., 2018; Zhou H.J. et al., 2018), lncRNAs GAS5 has an inhibitory effect on M2 polarization of microglia and increases demyelination (Sun et al., 2017).

## Dna Methylation

DNA methylation is a chemical chromatin modification occurring on the cytosine residues from CpGs dinucleotides. DNA methylation leads to repression of gene transcription when CpG islands (regions of the genome containing high density of CpGs) are methylated (Goll and Bestor, 2005). DNA methylation corresponds to the addition of a methyl group on the targeted cytosine from a CpG dinucleotide. Once methylated, the cytosine is referred as 5-methyl cytosine or 5-mC. During gametogenesis and embryogenesis, DNA methylation plays a major role in regulating the chromatin organization and the expression of genes. (Goll and Bestor, 2005; Surani et al., 2008). DNA methylation patterns are regulated by specific enzymes called DNA methyltransferases or DNMTs which can act as *de novo* or maintenance DNA methylation enzymes.

De novo DNA methyltransferases add methyl groups after the replication of the DNA while maintenance methyltransferases will act on hemi-methylated DNA during the replication of the DNA (Prokhortchouk and Defossez, 2008). Ten–eleven translocase (TETs) family enzymes catalyze 5-mC marks to 5-hmC (5-hydroxymethylcytosine), which corresponds to an active demethylation process leading to an increase of gene expression (Tahiliani et al., 2009). DNA methylation is involved in different cellular processes. Indeed, DNA methylation regulates the X chromosome inactivation, the silencing of centromeric and repetitive sequences but also mammalian imprinting showing the importance of DNA methylation in term of stable and heritable epigenetic regulation (Bestor, 2000; Li, 2002; Reik, 2007).

In contrast to the extensive investigations made on the effect of HDACs inhibitors on microglial gene expression, the regulation of gene expression by DNA methylation is poorly studied in those cells. Two different approaches have been used to investigate the impact of DNA methylation in microglia. This epigenetic modification has been investigated either at the global level, looking for changes in total DNA methylation, or at selected targeted sites, looking at specific gene methylation and related gene expression.

Few studies focused on the global level of DNA methylation in the brain associated to specific brain injury or disease. By immunochemistry, a global hypermethylation has been observed in AD brain and also a significant increase of 5-hmC in the middle frontal gyrus and middle temporal gyrus of human AD brain (Coppieters et al., 2014). The authors also observed that the levels of 5-mC and 5-hmC were low in microglia in control and AD brains. This confirms the observation made by Phipps et al. (2016) whom have shown no differences in 5-mC or 5-hmC in AD in microglia or interneurons. Moreover, no differences were observed in 5-mC or 5-hmC in cells in plaque free regions or near the plaque in late AD (Phipps et al., 2016). So even if AD is associated with microglia activation, it seems that global DNA methylation changes occurring during the disease need to be investigated further in order to confirm a role of DNA methylation in microglia. On the opposite, global methylation changes in microglia has been observed in a rat model of traumatic brain injury (TBI). In this model, by using an immunohistochemistry and a double staining approaches, a sub-population of reactive microglia has been identified and this population is characterized as the major source of hypomethylated cells (Zhang et al., 2007).

When focusing on specific gene methylation, it has been shown that *IL1β* gene expression is regulated by DNA methylation in aging microglia. Indeed, *IL1β* gene hypomethylation is associated with upregulation of the cytokine production in two different models of aging. In the first one, the authors associated SIRT1 deficiency to an upregulation of *IL1β* by hypomethylation of specific CpGs sites on *IL1β* proximal promoter (Cho S.H. et al., 2015). Matt et al. (2016) confirmed that *IL1β* upregulation is due to its hypomethylation and validates the role of DNA methylation by treating BV2 and primary microglia cells with the DNA methylation inhibitor 5-azacytidine which increased *IL1β* gene expression.

In AD, S-Adenosylhomocysteine (SAH), is a potent inhibitor of methyltransferases. SAH increases the production of amyeloid-β in BV2 microglia cells possibly by increasing the expression of amyloid precursor protein (APP) via the promotion of hypomethylation of the *APP* and *PS1* gene promoters (Lin et al., 2009). Although SAH only significantly increased the expression of BACE1 (beta-site APP cleaving enzyme 1) at the highest concentration used, Byun et al. (2012) uncovered two specific methylation sites of *Bace1* in BV2 microglia cells. By using a bisulfite sequencing approach, they showed that after treatment of BV2 cells with 5-Azacytidine, the *Bace1* gene presented specific demethylation of two CpG sites (+298 and +351) in its 5′-UTR region. Mitochondrial dysfunction has been implicated in the pathogenesis of PD. Indeed, in brain tissue from PD patients, a reduction of the expression of PGC-1α (peroxisome proliferator-activated receptor gamma coactivator-1) has been observed. Su et al. (2015) investigated the *PGC-1α* promoter methylation after activation of neuroinflammation by the pro-inflammatory acid palmitate. They observed that palmitate induces *PGC-1α* promoter methylation in microglia cells as well as mouse primary cortical neurons and astrocytes which reduces the expression of the gene and reduces the mitochondrial content (Su et al., 2015). Finally, it is shown that a specific DNMT is involved in microglia activation. Indeed, by transcriptome sequencing approaches, DNMT3L is found upregulated after microglia activation either by LPS treatment or by stimulation of microglia with TLR3 and TLR4 ligands (Das et al., 2015a,b).

## Perspectives

**FIGURE 2:**
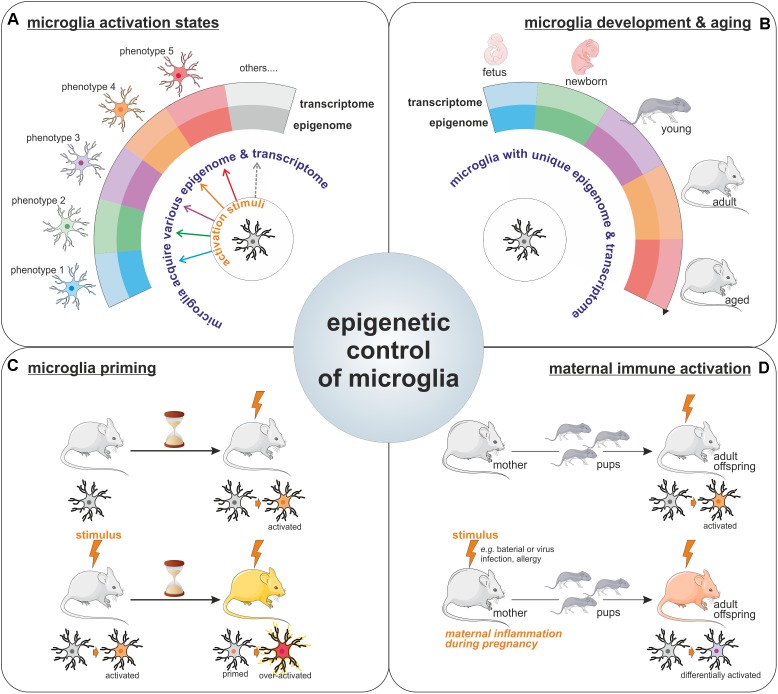
Epigenetic control of microglia. Illustration of the importance for epigenetics mechanisms for the acquisition of microglia activation states in response to various stimuli **(A)**, for the definition of unique microglial molecular signatures throughout life, including microglia development and aging (the different microglia colors represents the different phenotypes these cells can harbor like pro-inflammatory/wound healing/pro-tumoral phenotypes among others) **(B)**, as well as for the long-lasting effect observed on microglia phenotypes such as in the context of microglial priming **(C)** or upon maternal immune activation **(D)**.

For a long period of time, microglia have been considered as cells capable of polarization into two distinct phenotypes (so called M1 and M2 phenotypes), however, nowadays it is clear that microglia can multiple phenotypes and that the regulation of microglia activation is far more complicated than initially described. The transcriptional and epigenetic machineries have emerged as important regulators of microglia phenotype acquisition (**Figure 2A**). Indeed, specific transcriptome profiles have been shown to define the distinct microglial phenotypes acquired in response to various stimuli or challenges with the CNS (Holtman et al., 2017). Collectively the above-mentioned reports strongly support the contribution of epigenetic mechanisms to the acquisition of these different microglial activation states. Worth a notice, even in the context of the unchallenged brain, microglia exhibit different transcriptome throughout life (Butovsky et al., 2014; **Figure 2B**). Moreover, surveying microglia as well as activated ones can be characterized by distinct miRNA signatures (Butovsky et al., 2014). Thus, it seems that unique epigenome and transcriptome can define the different microglia states of activation in the developing, aging, and diseased brain. Furthermore, a recent study illustrated the idea that since microglia possess different epigenomes and associated transcriptomes throughout life, and in the course of diseases, intervention of the epigenetics machinery could therefore have different impacts on these cells. In fact, *Hdac1* and *Hdac2* depletion in microglia led to contrasting effects in the developing, the homeostatic and the diseased brain (Datta et al., 2018).

The involvement of epigenetic mechanisms in the control of microglia polarization toward a specific phenotype or activation state raises the question; how long microglia can be affected by such epigenetic changes? Since microglia are capable of both innate and adaptive responses, it has been proposed that they can acquire a memory of particular events. Indeed, there are compelling evidences for a microglial epigenetic memory, which is illustrated by the observed differential response to a challenge by microglia, which have been previously exposed to the same or a different challenge, as compared to the response of naive microglia. This process is known in the field as the microglia priming (**Figure 2C**). For example, priming of microglia with a LPS stimulus leads to a different response to a second stimuli (Liu et al., 2012). This epigenetic memory of primed microglia has been confirmed in a model of long-lasting pro-inflammatory suppression where the microglia presents an immune-suppressed phenotype acquired by epigenetic changes after LPS-preconditioning (with a reduction in H3K4me3 on *IL1-β* and *TNF-α* promoters) to prevent excessive damages (Schaafsma et al., 2015). This acquired memory by microglia after priming is also observed *in vivo* in the case of development and aging where *IL10* gene, for example, is specifically regulated by methylation in the adult after an early life drug experience (Schwarz et al., 2011). Epigenetic regulation is also involved in microglia memory during development since pre-exposed microglia to LPS *in vivo* shown different patterns of gene expression including HDACs prior and after a second exposure to LPS *in vitro* (Cao et al., 2015). Recently, immune memory of microglia has been observed *in vivo* after inflammatory stimuli. In their model, Wendeln and coworkers observe that priming of microglia leads to an acute immune training and tolerance in the brain, that microglia are reprogrammed epigenetically and that these changes could be involved in differential responses to neuropathology (Wendeln et al., 2018).

It is known that epigenetic modifications are heritable marks like DNA methylation and can be sustained in time but also into generations. This brings the point on a long term regulation of microglia by epigenetic modifications or signatures. It is very interesting to observe the heritable phenotypes of microglia through the generations. In mammals, maternal immune activation, or MIA, which can be due to an inflammatory stimulus like bacterial or viral infections but also activated after allergy during the pregnancy, has been shown to positively correlates with the risk of developing neuropsychiatric disorders like autism in the offspring (Vogel Ciernia et al., 2018). This link may be bridged to an impact of MIA on the microglia from the fetus which leads to the acquisition of functional changes that are maintained in the adult (Mattei et al., 2017; Prins et al., 2018; **Figure 2D**). In a majority of animal models of maternal immune activation, the rat dams or pregnant mice are treated with either a viral mimetic (poly I:C) or LPS. Microglia in offspring from MIA challenged rodent show an altered transcriptome signature, with increased expression of genes related to pro-inflammatory signaling pathways, but a reduction in expression of genes related to proliferation and cell cycle (Ben-Yehuda et al., 2017; Mattei et al., 2017). The transgenerational effect of MIA, has led to the proposal that microglia epigenetic changes, that can induce long-term modification of microglia phenotype, should be responsible for the observed effect in progenies (Nardone and Elliott, 2016). Indeed, recent study shows alteration of the microglial DNA methylome in a mouse asthma model of MIA. Genome wide analysis identified alteration in the expression of genes involved in the control of microglial sensitivity to the environment and in the shaping of neuronal connections in the developing brain (Vogel Ciernia et al., 2018).

Finally, one should keep in mind that it is highly improbable that the control of microglia plasticity is limited to epigenetic mechanisms. For example, metabolic changes in the microglia and its microenvironment have been linked to microglia phenotype polarization and associated to various diseases (Gimeno-Bayon et al., 2014; Kalsbeek et al., 2016; Orihuela et al., 2016). Thus, there is a multifactorial regulation of microglia activation states, and this level of complexity should be taken into account when designing potential therapeutic strategies targeting microglial epigenetic mechanisms.

## Author Contributions

MC and BJ planned and wrote the manuscript.

## Conflict of Interest Statement

The authors declare that the research was conducted in the absence of any commercial or financial relationships that could be construed as a potential conflict of interest. The reviewer RH declared a shared affiliation, with no collaboration, with the authors to the handling Editor.
